# Risk of Exposure to Steviol Glycosides Through Consumption of Foods and Beverages: A Survey of the Thai Population

**DOI:** 10.1002/fsn3.4681

**Published:** 2025-01-09

**Authors:** Pharrunrat Tanaviyutpakdee, Pimpuk Phitwongtaewan, Yollada Phungsiangdee, Weeraya Karnpanit, Pranee Phattanakulanun, Thipaporn Yomvachirasin, Songsak Srianujata

**Affiliations:** ^1^ Food Toxicology Division, Institute of Nutrition Mahidol University Nakhon Pathom Thailand; ^2^ Master of Science Program in Toxicology and Nutrition for Food Safety, Institute of Nutrition Mahidol University Nakhon Pathom Thailand; ^3^ School of Molecular and Life Sciences Curtin University Bentley Western Australia Australia; ^4^ Thailand Risk Assessment Center, Institute of Nutrition Mahidol University Nakhon Pathom Thailand

**Keywords:** consumption survey, estimated daily intake, marketed products, risk assessment, stevia

## Abstract

This study aimed to assess dietary exposure to steviol glycosides and identify the key contributors to the exposure in the Thai population. In total, 2114 participants were included in this study, and a semiquantitative food frequency questionnaire and a photobook of foods and beverages were used to collect their food consumption data. In addition, the body weight data of the participants were recorded, and a survey was conducted to obtain information regarding their consumption of foods and beverages that contain steviol glycosides, including data on the amount consumed and the frequency of intake. The amount of steviol glycosides in the foods and beverages was determined using ultrahigh‐performance liquid chromatography‐electrospray ionization tandem mass spectrometry. We used data on the mean and 97.5th percentile (PCTL) consumption of food or beverages for both the per capita and consumer‐only scenarios to estimate the dietary intake of steviol glycosides from consumption of food and beverages. The risk of steviol glycoside exposure was evaluated using the hazard quotient, which involved a comparison of the estimated dietary intake with the acceptable daily intake (ADI) of steviol equivalents (4 mg/kg BW/day). Children aged 3–9 years had the highest mean and 97.5th PCTL intake per capita, which accounted for 2.35% and 22.95% of the ADI, respectively. The results of this study demonstrate that the estimated intake of steviol glycosides by the Thai population across all scenarios and age groups is below the ADI, indicating that foods and beverages that contain steviol glycosides do not pose a risk to the Thai population. Nevertheless, the top three contributors to the intake of steviol glycosides, which are products in categories 14 (beverages), 11 (sweeteners), and 1 (dairy products and analogues), should be consumed cautiously.

## Graphical abstract

From the market survey, 192 products declared the use of steviol glycosides among 1285 products that declared the use of intense sweeteners on their labels. Most products met regulatory limits for steviol glycoside concentrations, except for four beverages. Category 14 Beverages, category 11 Tabletop sweeteners, and category 1 dairy products and analogues were the top three contributors to steviol glycoside intake. The intake of steviol glycosides in Thai population was below the ADI across all age groups.

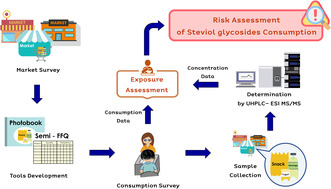

## 1 Introduction

Excessive intake of sugar‐sweetened foods and beverages is associated with an increased risk of non‐communicable and metabolic diseases, including obesity, diabetes, cardiovascular diseases, and cancer (World Health Organization (WHO) 2015). The WHO recommends reducing the consumption of free sugars to < 10% of the total energy intake, which is approximately equivalent to 12 teaspoons of sucrose for adults. Further reduction to < 5%, or approximately 25 g (6 teaspoons) per day, will provide additional health benefits (World Health Organization (WHO) 2017). Thailand implemented tax rates on sugar‐sweetened beverages in 2017 (Phonsuk et al. 2021). Notably, the increased awareness of reduced sugar consumption for improved health has led to increased replacement of sucrose with low and no‐calorie sweeteners (LNCSs) in foods and beverages (Phonsuk et al. 2021).

Some previous studies have demonstrated that LNCSs are associated with some adverse effects, including increased intestinal absorption of glucose and fructose, alterations in the composition of the intestinal microbiota, and increased risk of developing obesity and type 2 diabetes, health authorities have expressed concerns regarding the increase in their consumption (Schiano et al. 2021). The food industry has shifted its focus from synthetic substances to natural sources of LNCSs such as tagatose, thaumatin, and particularly, steviol glycosides (Saraiva et al. 2020). Due to concerns regarding the adverse effects of sugars and synthetic LNCSs, steviol glycosides and their derivatives are increasingly being used in food production worldwide (Ciriminna et al. 2019). Thai Food and Drug Administration has endorsed the use of steviol glycosides by the Notification of Ministry of Public Health No. 444 B.E. 2566 (2023), allowing their use in 14 food and beverage categories. The FAO/WHO Joint Expert Committee on Food Additives (JECFA) and the European Food Safety Authority (EFSA) have established an acceptable daily intake (ADI) for steviol glycosides, expressed as steviol equivalents, of 4 mg/kg bw/day. The EFSA Panel on Food Additives and Nutrient Sources Added to Food (ANS) conducted an exposure assessment of steviol glycosides based on maximum use levels. The results from the conservative estimates of steviol glycoside exposures in both adults and children indicate that the exposures are likely to exceed the ADI if calculated using the maximum proposed use levels (European Food Safety Authority (EFSA) 2010).

Regarding safety considerations, exposure to food additives is routinely monitored based on the principle of risk assessment. The tiered approach includes three tiers: Tiers 1, 2, and 3. Tier 1 involves the assessment of a combination of theoretical food consumption data and the maximum permitted usage levels of the additive. Tier 2 assessment involves the evaluation of a combination of actual food consumption data and the maximum permitted usage levels of the additive. Tier 3 is the assessment of a combination of actual food consumption data (from national or specific surveys) and the actual usage levels of the additive (European Commission (E.C.) 2001). A study conducted in Belgium, which involved a Tier 2 assessment of dietary intake of steviol glycoside, revealed that the high‐intake child consumers exceeded the ADI for steviol glycoside. In Asia, the data regarding exposure of the general population to steviol glycosides is limited. A review of the existing literature revealed that 16 studies on the daily intake of LNCSs among Asians were conducted between 2008 and 2017. Of these, four focused on steviol glycoside exposure (Martyn et al. 2018). In Australia and New Zealand, the average and 90th percentile steviol glycoside intakes of non‐ ‘brand loyal’ consumers in all population groups were up to 60% of the ADI for steviol glycosides. The ‘brand loyal’ consumers exceeded the 90th percentile intake (up to 110% for children aged 2–6 years) (Food Standards Australia New Zealand (FSANZ) 2011). Six of 26 studies conducted in Europe focused on exposure to steviol glycoside (Martyn et al. 2018). For EU members, the maximum intake among ‘brand loyal’ toddlers marginally exceeded the ADI (Tennant and Bruyninckx 2018).

Steviol glycosides are added to various foods and beverages sold in Thailand, potentially resulting in a daily intake higher than the ADI. However, data on the risk of exposure to steviol glycosides in foods and beverages in Thailand is lacking. Therefore, this study was conducted to estimate the risks associated with the consumption of foods and beverages that contain steviol glycosides and analyze possible concerns regarding steviol glycoside intake in the Thai population. The data from this study could be used for the adjustment of the maximum steviol glycoside intake level stated in NMPH No.444 B.E. 2566 (2023), as notified by the Ministry of Public Health. Furthermore, the data obtained from this study can be used in interactions with the representatives of the food industry during public hearings for the development of recommendations regarding consumer safety in the consumption of both local and exported products.

## 2 Materials and Methods

We commenced this study by conducting a market survey to obtain relevant information for the creation of tools for collecting the food consumption and personal data of the participants. After the food consumption data were collected, the food and beverage types were ranked, and representative samples were analyzed to measure the steviol glycoside concentrations in the food items. The details of each of the abovementioned steps are described below.

### 2.1 Market Survey

We conducted a market survey between July 2020 and October 2020 to identify a variety of food and beverage products that contain steviol glycosides. The dietary information and ingredients printed on the packaging of identified products were assessed and recorded. The data obtained from this survey were then used to create a tool for the food consumption survey. The categories and types of foods and beverages outlined in NMPH No. 444 B.E. 2566 (2023) were used as references for the product checklist used during the market survey. Under the NMPH No. 444 regulation, the Thai Food and Drug Administration permitted the use of steviol glycosides in 14 categories (51 subgroups) of foods and beverages. The maximum usage levels for the categories range from 30 to 2500 mg/kg and comply with the Good Manufacturing Practice standards in some categories. The categories of foods and beverages to which steviol glycosides may be added, according to NMPH No. 444, are outlined in Table 1. Representative food retailers were selected from Thai retail food markets (Statista 2023), including Big C, Tesco Lotus, Tops, Gourmet Market, Central Food Hall, Villa Market, and Foodland, as well as grocery stores such as 7‐Eleven and Family Mart located in the Bangkok and Nakhon Pathom provinces. The survey of products that contain steviol glycosides and their derivatives sold by 33 food retailers is illustrated in Figure S1.

**TABLE 1:** The categories of foods and beverages with steviol glycosides and their derivatives.

Category	Maximum level (mg/kg)
01. Dairy products and analogs	70–330
02. Fats and oils, and fat emulsions	330
03. Edible ice	270
04. Fruits, vegetables, seaweeds, nuts, and seeds	40–350
05. Confectionery and chewing gum	700–1100
06. Cereals and cereal products	100–350
08. Meat and meat products	100
09. Fish and fish products	100–165
10. Eggs and egg products	330
11. Tabletop sweeteners (including honey)	GMP
12. Salts, spices, soups, sauces, salads, and protein products	30–350
13. Foodstuffs intended for specific nutritional uses	270–2500
14. Beverages	115–200
15. Ready‐to‐eat savories	170

Abbreviation: GMP, Good manufacturing practice.

### 2.2 Preparation of the Interview Tools

#### 2.2.1 Photobook

A photobook was created to assist consumers in reviewing the food and beverage information collected during the market survey. Assessment of the nutritional information and ingredients outlined on the packaging of the products indicated that 1285 products included at least one type of intense sweetener in their lists of ingredients. Of these, 192 contained steviol glycosides. These 192 products were purchased to be photographed for the creation of the photobook and questionnaire for the next step. As shown in the Figure S2, photographs of the foods or beverages in the photobook were arranged according to the categories outlined in NMPH No. 444 B.E. 2566 (2023).

#### 2.2.2 Questionnaire

The questionnaire was divided into two parts: one section for obtaining the general information of the participants and the other for collecting food consumption data.

##### 2.2.2.1 Part I: General Information of the Participants

The questions included in this section of the questionnaire (Figure S3) were questions on the general information of the participants, including their first and last names, weight, height, and age. Additionally, this section included questions related to knowledge, attitudes, and practices regarding the consumption of foods and beverages that contain steviol glycosides and their derivatives, as well as other types of LNCSs.

##### 2.2.2.2 Part II: Semi‐Quantitative Food Frequency Questionnaire

A semiquantitative food frequency questionnaire (semi‐FFQ) (Figure S3) was designed to collect food consumption data. Food and beverage items were arranged on a table in the semi‐FFQ, and a corresponding picture of each item was included in the photobook. The participants were asked about the amount of the item consumed and frequency of consumption; hence, the quality of the data obtained depended on the participants' recall ability. Therefore, the photobook served as a valuable tool to assist the participants in recalling food and beverage consumption data. The amount of each food or beverage consumed was estimated based on “A,” the reference portion or serving size of each item. Quantities that exceeded “A” were estimated to be 2A and 3A, whereas smaller quantities were estimated to be ½ A and ¼ A. The reference portion sizes for foods and beverages were obtained from the data on the portion or serving size indicated on the packaging of each item and from the Notification of the Ministry of Public Health No. 182 B.E. 2541 (1998). The reliability and validity of the developed tools, including the photobook and semi‐FFQ, were assessed (Browne 1995). Thirty volunteers (aged 3–≥ 60 years) were recruited for interviews conducted at the Institute of Nutrition, Mahidol University, using the developed tools. A Cronbach's alpha of 0.90 was obtained after the assessment, indicating excellent reliability of the data collected using this questionnaire. After acquiring food consumption data from the survey, the exact amount of each type of food and beverage consumed was determined by multiplying the amount consumed by the frequency of consumption.

### 2.3 Exposure Assessment

The Tier 3 approach was employed in this study to calculate exposure to steviol glycosides and their derivatives. The food consumption survey data included the amounts of food consumed and the body weights of the participants. The concentrations of steviol glycoside in the food items were determined using ultrahigh‐performance liquid chromatography–electrospray ionization tandem mass spectrometry (UHPLC–ESI‐MS/MS).

#### 2.3.1 Consumption Survey

Prior to the collection of food consumption data, the study protocols and procedures were approved by the Committee for Research Ethics (Social Sciences), Faculty of Social Sciences and Humanities, Mahidol University (MUSSIRB No. 2020/093 (B1)) on February 29, 2020. Mothers of children aged < 6 years provided written informed consent on their behalf. The interviewers underwent training to familiarize themselves with the questionnaire, photobook interview tools, and other related documents. Cochran's equation (Cochran 1977) was used to calculate the size of the study population, as shown in Equation (1).

(1) $$n=\frac{Z^{2}p\left(1-p\right)}{e^{2}}$$

$$n=\frac{{\left(1.96\right)}^{2}0.48\;\left(1-0.48\right)}{0.05^{2}}$$

n=384persons/province

where ***n*** is the required number of participants, *Z* = 1.96 represents the value from the standard normal distribution, reflecting a confidence level of 95%, and *e* = 0.05 denotes the desired level of precision or the margin of error at 95%. *p* and *q* (which is 1−*p*) represent the proportion of the population that consumes and does not consume the products (that contain intense sweeteners), respectively. In this study, *p* was set at 0.48, a value that was obtained from a previous study (Tanaviyutpakdee et al. 2021).

A sample size of 384 persons per province obtained from the equation was adjusted for a 10% non‐response rate, resulting in a sample size of 422 participants per province. This study was conducted in five provinces: Bangkok, Ayutthaya, Chiang Mai, Ubon Ratchathani, and Songkla, representing the capital, central, northern, northeastern, and southern parts of Thailand, respectively. Therefore, the total number of Thai participants included in the present study was 2114. The number of participants in each age group was calculated based on the proportions of the age groups in each province. A photobook and a validated questionnaire were used for the collection of food consumption data. The participants were categorized into five age groups according to the age groups used by the National Bureau of Agricultural Commodity and Food Standards (Ministry of Agriculture and Cooperatives 2016): children (age 3–9 years), teenagers (age 10–18 years), young adults (age 19–39 years), adults (age 40–59 years), and individuals aged 60 or above (≥ 60 years).

#### 2.3.2 Concentration of Steviol Glycosides

Food and beverage samples were selected based on the consumption patterns observed in the food consumption survey. Products that were consumed by one or more participants were selected as representative samples, and the concentrations of steviol glycosides and their derivatives in the samples were determined. Random lots of products were purchased from three retail markets and combined into pooled samples of each type of food or beverage. Analysis of steviol glycosides and derivatives was conducted using UHPLC‐ESI‐MS/MS (Thermo Fisher Scientific Ultimate 3000 System, Burladingen, Germany). This system comprises an HPG‐3200SD high‐pressure gradient pump, a WPS‐3000 thermostatized split‐loop autosampler, and a TCC‐3000 RS column thermostat. The analysis of steviol glycosides and their derivatives was performed according to the previous method described by Phungsiangdee et al. (2023). Briefly, to determine the concentration of steviol glycosides in a selected item, 1 g of a well‐mixed sample of the item was dissolved in 5 mL of deionized water and acetonitrile (80:20 v/v) and sonicated in an ultrasonic bath for 10 min. The volume was adjusted to 10 mL and filtered through a Whatman No. 1 filter paper. For milk‐based samples, 2 g of the homogenized sample was dissolved in 1.5 mL of deionized water and vortexed for 1 min. The suspension was then incubated at 60°C for 10 min and allowed to cool to room temperature before 2 mL each of Carrez I and Carrez II and 1 mL of acetonitrile were added to it. The volume of the solution was adjusted to 10 mL using deionized water and vortexed for 1 min. The solution was maintained at room temperature for 15 min and centrifuged at 4000 rpm for 1 h. The supernatant was collected and filtered through a Whatman No. 1 filter and cleaned using solid‐phase extraction (SPE).

For high‐fat samples, 1 g of the homogenized sample was dissolved in 5 mL of water with acetonitrile (80:20 v/v) and sonicated for 10 min. Subsequently, 1 mL each of Carrez I and 1 mL of Carrez II were added to the solution. The volume was adjusted to 10 mL using water and acetonitrile (80:20, v/v) and vortexed for 1 min. The solution was maintained at room temperature for 15 min and centrifuged at 4000 rpm for 1 h. The supernatant was then filtered through a Whatman No. 1 filter paper and cleaned using SPE. The prepared solutions of all sample types were filtered through a 0.2 μm polytetrafluoroethylene (PTFE) membrane before being subjected to UHPLC‐ESI‐MS/MS.

The concentration of each steviol glycoside derivative was calculated as a steviol equivalent by multiplying the value obtained by the factors provided in the Table S1. Percentage recovery (% recovery) and percentage relative standard deviation (% RSD) were used to ensure the accuracy and precision of the analysis results, respectively.

#### 2.3.3 Calculation of Exposure to Steviol Glycosides or Estimated Daily Intake

A Tier 3 deterministic exposure assessment was performed according to the WHO guidelines (2021) to estimate exposure to steviol glycosides as steviol equivalents from the consumption of foods and beverages (Equation 2). The average and 97.5th percentile (PCTL) consumption of food or beverage for both the per capita and consumer‐only scenarios, along with the actual or average concentration of steviol equivalents, were considered for the calculation of exposure in four scenarios, as demonstrated below.

(2) $$\mathrm{EDI}\;\left(\mathrm{mg}/\mathrm{kg}\;\mathrm{bw}/\mathrm{day}\right)=\frac{\text{Amount consumed}\;\left(\mathrm{kg}/\mathrm{day}\right)\times \text{Concentration of steviol equivalent}\;\left(\mathrm{mg}/\mathrm{kg}\right)\;}{\text{Actual body weight of participant}\;\left(\mathrm{kg}\;\mathrm{bw}\right)}$$

EDI, estimated daily intake.

Per capita (consumers + non‐consumers, i.e., total population):

Scenario 1: Average exposure per capita was calculated by multiplying the average consumption per capita by the actual or mean concentration.

Scenario 2: High‐level exposure per capita (97.5th PCTL consumption per capita × actual or mean concentration).

Consumer only (consumer only, i.e., total population of consumers, excluding non‐consumers):

Scenario 3: Average consumer‐only exposure (average consumption by consumers only × actual or mean concentration).

Scenario 4: High‐level consumer‐only exposure (97.5 PCTL consumption by consumers only × actual or mean concentration).

### 2.4 Characterization of the Risk of Exposure to Steviol Glycosides

The hazard quotient (HQ) was used to assess the risk associated with exposure to steviol glycosides by comparing the exposure data with the ADI of 4 mg/kg bw/day for steviol equivalents. Characterization of the risk of steviol glycoside exposure due to the consumption of food and beverages that contain LNCSs was performed as the HQ (Equation 3). The risk is reported as a percentage of the ADI, which was obtained by multiplying the HQ by 100. An estimated daily intake (EDI) that exceeded the ADI (HQ is > 1 or > 100% of the ADI) was considered indicative of a risk associated with the consumption of steviol glycosides.

(3) $$\mathrm{HQ}=\frac{\text{Exposure to sweetener}}{\mathrm{ADI}\;\text{of each sweetener}}$$

### 2.5 Statistical Analysis

**FIGURE 1:**
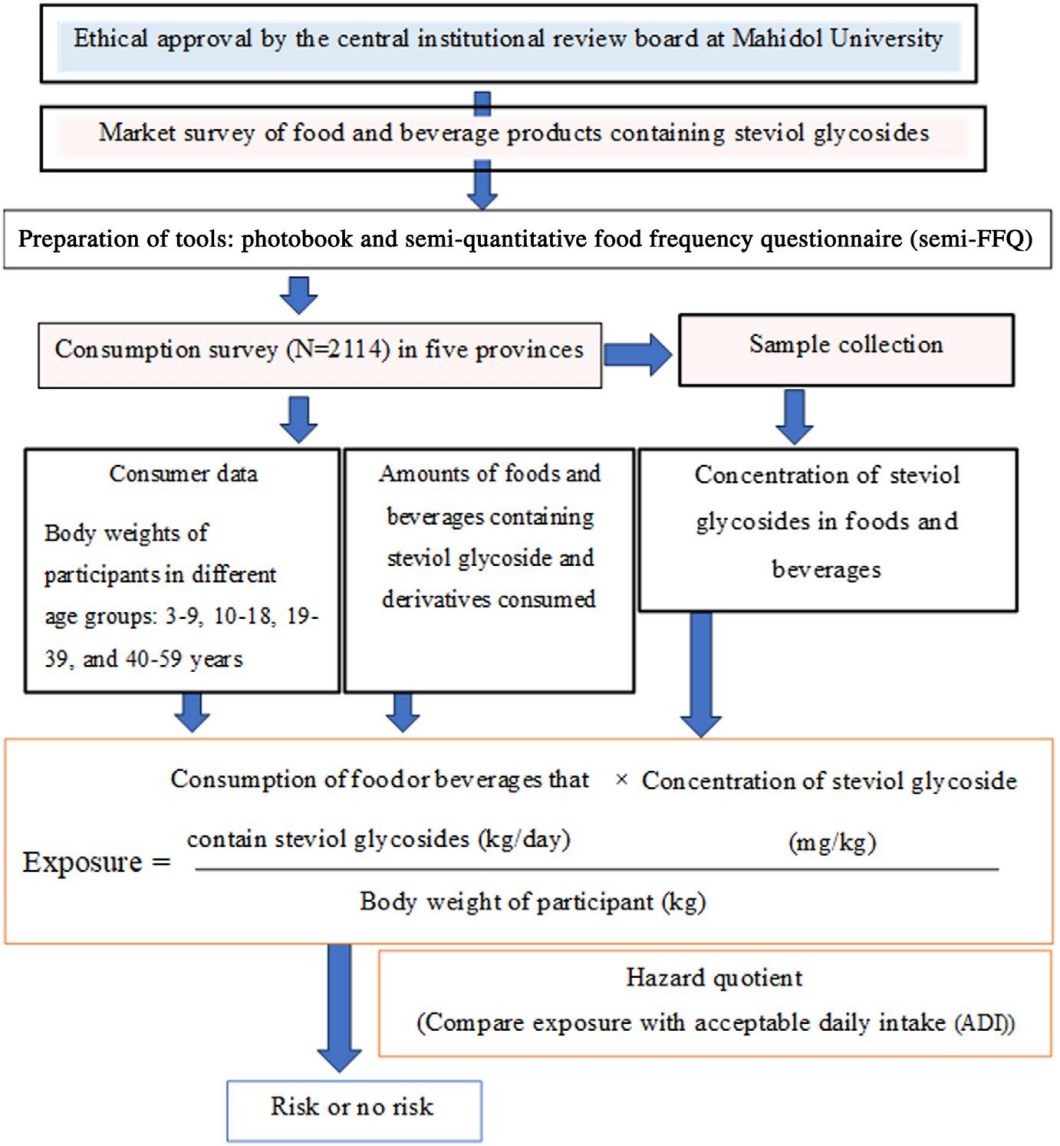
Conceptual framework of the study.

Estimated daily intake or exposure to steviol glycosides is expressed as mg/kg bw/day. The mean, standard deviation (SD), and 97.5th PCTL of intake were calculated for the total population (per capita) and for consumers only. Data were analyzed using PASW Statistics software (formerly SPSS Statistics) version 19 (IBM, Chicago, Il, USA). The conceptual framework of this study is presented in Figure 1.

## 3 Results and Discussion

### 3.1 Products That Contain Steviol Glycosides

**FIGURE 2:**
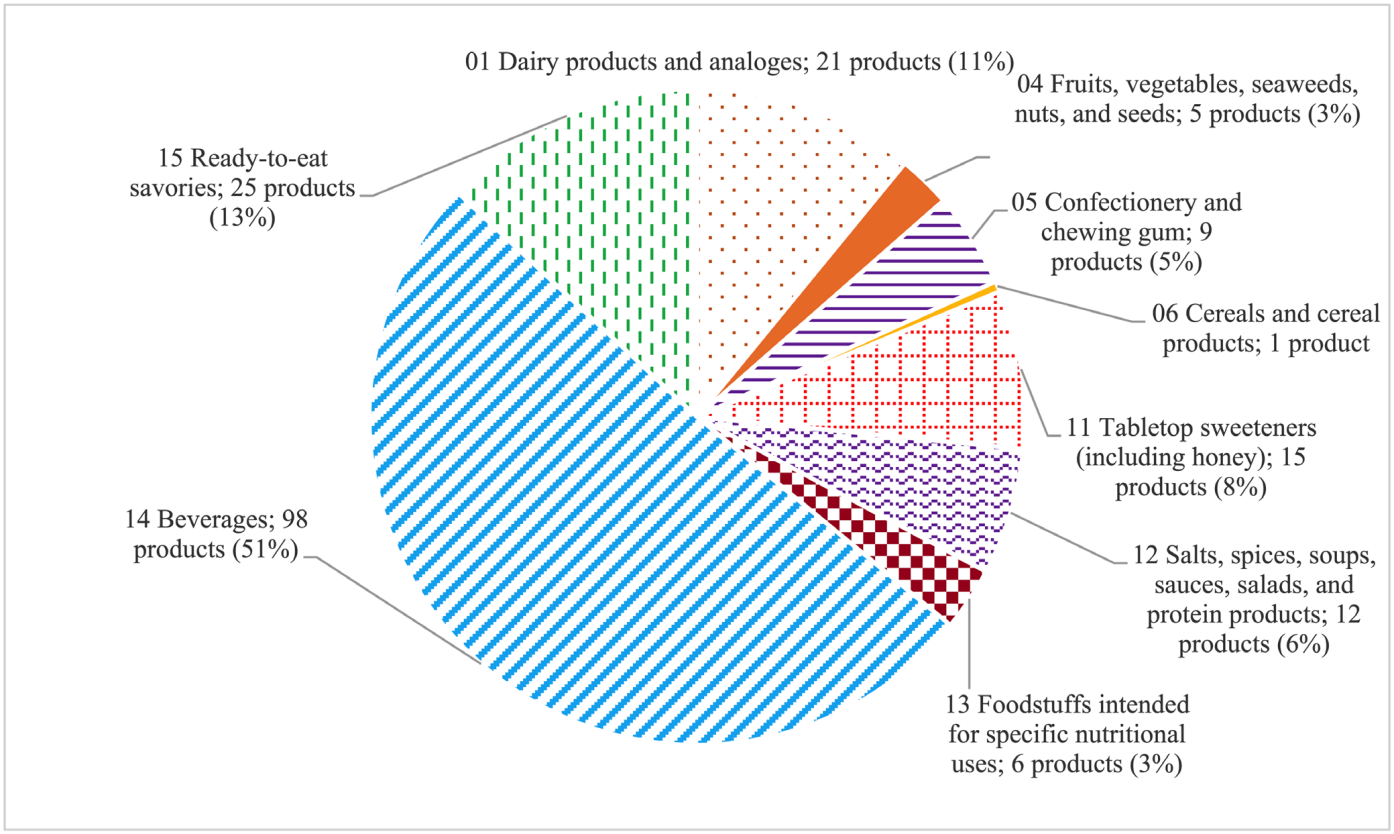
Products with steviol glycosides listed as ingredients on their labels.

The products that contain steviol glycosides and their derivatives identified in the markets were classified according to the product categories specified in NMPH No. 444 B.E. 2566 (2023). According to the regulation, use of steviol glycosides as sweeteners is allowed for foods and beverages in 14 categories. A review of product labels indicated that 192 products contained steviol glycosides. These 192 products were classified into nine categories of foods and beverages. The category with the highest number of products was category 14, which is beverages, excluding dairy products (51%, 98 out of 192 products). Category 15, ready‐to‐eat savories, had the second‐highest number of products (13%, *n* = 25). The category with the third highest number of products was category 1, dairy products and analogs (11%, *n* = 21) (Figure 2). Although the categories of foods and beverages adopted in other studies are not identical to those used in the present study, the distribution of the main categories in this study is similar to those reported in several previous studies. For instance, in a study conducted in Chile by Sambra et al. (2020), 38.2% of the food items consumed by the participants were non‐alcoholic beverages, 28.8% were dairy products, 15.6% were sweets and other desserts, 14.5% were cereal products, and 2.9% were processed fruits. The sequence of the food categories reported in a Spanish study differed from that in our study, particularly for categories 14, 15, and 1. However, the authors of the Spanish study also ranked the top three out of five categories of products that contained steviol glycosides (Samaniego‐Vaesken et al. 2021). In an Italian study (Le Donne et al. 2017), a survey of the labels on 326 products that contain LNCSs indicated that non‐alcoholic beverages, tabletop sweeteners, and food supplements were the major contributors to exposure to almost all types of sweeteners in the study population. In a study conducted in Brazil by Barraj et al. (2021), steviol glycosides were detected in processed foods and beverages such as diet cakes and cookies, fruit drinks, hot cocoa mixes, yogurt drinks, and tabletop sweeteners. Lenighan et al. (2023) reported that in their study, the highest usage of steviol glycosides as sweeteners was observed in products in category 14.1.4, which includes flavored water and drink products in Canada (100%) and the United States (67%) and ready‐to‐drink tea in Mexico (89%). Reports from Australia and New Zealand indicated that 123 and 109 products, respectively, contain steviol glycosides. These products are mainly in categories 14 (beverages), 7 (biscuits, crackers, cakes, pastries, and scones), 20 (gravy, sauces, and condiments), and 1 (liquid milk products and flavored liquid milk) (Food Standards Australia New Zealand (FSANZ) 2023). In the present study, all category 11 sweeteners (including honey) (8%; *n* = 15) were tabletop sweeteners that contain steviol glycosides. These products are available in liquid, solid, and powder forms. Similarly, a previous study indicated that the tabletop sweeteners sold in Chile consisted of 15 products in both liquid and solid forms (Sambra et al. 2020).

**FIGURE 3:**
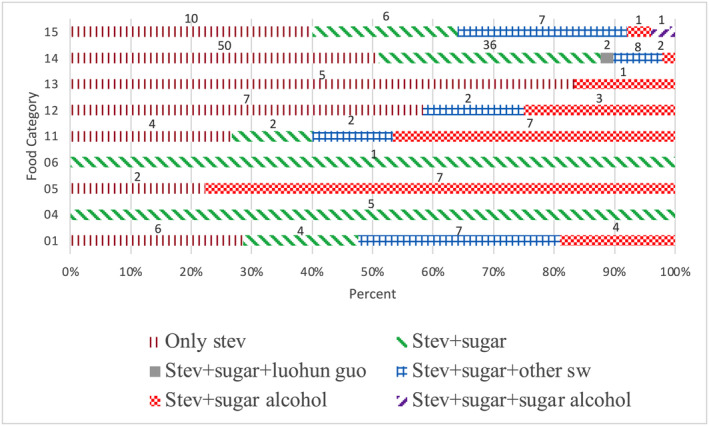
Patterns of the addition of sweeteners in food and beverages. SW, sweetener.

Information on the labels of the products indicated that they contained not only steviol glycosides but also other types of sweeteners, such as sugar, sugar alcohol, and other LNCSs. Six patterns of the addition of sweeteners in the products were observed: only steviol glycosides, steviol glycosides with sugar, steviol glycosides with sugar and luo han guo (monk fruit), steviol glycosides with sugar and other LNCSs (mainly acesulfame potassium, sucralose, and aspartame), steviol glycosides with sugar alcohol, and steviol glycosides with sugar and sugar alcohol. Eighty‐four products in category 14, accounting for approximately 44% of all the products, contained only steviol glycosides. The products that contain steviol glycosides spanned almost all categories except categories 4 and 6. One hundred and eight products, which constituted approximately 56% of all the products and were distributed across all categories, contained a combination of steviol glycosides and other sweeteners. The patterns of the addition of steviol glycosides in combination with other sweeteners are illustrated in Figure 3. Generally, LNCSs can be used alone or in combination with other sweetening agents. The practice of combining sweeteners has become a prevailing trend in the food industry because numerous additives impart secondary flavors, such as a bitter aftertaste, thereby constraining their application in the production of foods and beverages. The combination of various sweeteners can reduce or eliminate these undesirable residual flavors while enhancing the sweetness of the finished product and reducing the likelihood of exceeding the ADI (Zygler, Wasik, and Namie'snik 2010).

### 3.2 Demographic Data of the Participants

**FIGURE 4:**
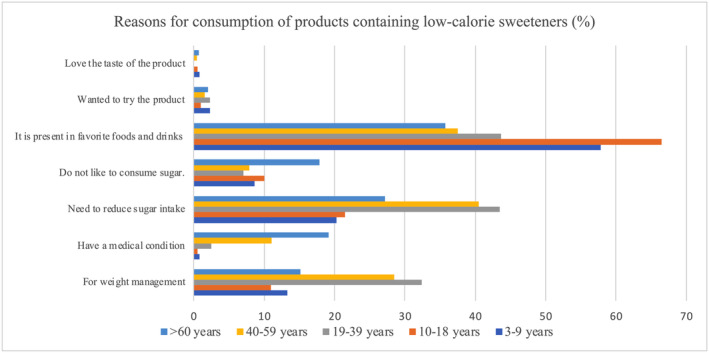
Reasons for consumption of products that contain steviol glycosides.

In total, 2114 consumers, comprising 881 males (42%) and 1233 females (58%), were included in this study through random cluster sampling of participants from five regions of Thailand. Most of the participants had a bachelor's degree (29.8%, *n* = 629). The second and third most common educational levels were primary (24.6%, *n* = 520) and secondary education (20.7%, *n* = 437), respectively. Approximately two‐thirds of the participants reported an income between 8000 and 15,000 Baht per month (35.0%, *n* = 650). Table 2 shows the age, sex, body weight, and height of the participants. All parameters are expressed as mean ± SD. The body mass index (BMI) of the consumers was calculated, and the results showed that the BMI for the 10–18, 19–39, 40–59, and 60+ years age groups was 20.76 ± 5.42, 24.24 ± 5.50, 25.89 ± 6.79, and 24.9 ± 4.27 kg/m^2^, respectively. However, it should be noted that BMI alone does not accurately reflect body composition and cannot distinguish between fat and muscle in children (Vanderwall et al. 2017). Thus, BMI was not calculated for the 3–9 years age group. The actual body weight of each participant was used in the calculation of exposure to steviol glycosides. The reasons for consuming products that contain steviol glycosides were ranked from greatest to minimal consumption: they are present in favorite foods and drinks (bought and consumed without thinking), need to reduce sugar intake, weight management, do not like to consume sugar, have medical conditions, wanted to try the product, and love the taste of the product (Figure 4).

**TABLE 2:** The body weight, height, and body mass index data of the participants.

Age group (years)	Sex	*n*	Body weight (kg)	Height (cm)	Body mass index (BMI, kg/m^2^)
3–9	Male	104	25.26 ± 9.22	116.38 ± 16.48	NA
	Female	116	23.59 ± 8.41	118.09 ± 16.97	NA
	Total	220	24.38 ± 8.84	117.28 ± 16.75	NA
10–18	Male	124	56.65 ± 19.79	161.99 ± 13.16	21.3 ± 6.00
	Female	140	49.80 ± 14.21	155.85 ± 9.24	20.29 ± 4.82
	Total	264	53.02 ± 17.36	158.73 ± 11.64	20.76 ± 5.42
19–39	Male	263	72.95 ± 17.42	171.84 ± 6.80	24.67 ± 5.57
	Female	422	65.36 ± 15.02	159.13 ± 6.16	23.96 ± 5.45
	Total	264	65.48 ± 17.03	164.02 ± 8.91	24.24 ± 5.50
40–59	Male	259	72.41 ± 14.71	167.3 ± 8.59	26.16 ± 9.18
	Female	384	63.18 ± 11.90	156.68 ± 6.49	25.72 ± 4.51
	Total	643	66.9 ± 13.86	160.95 ± 9.05	25.89 ± 6.79
60+	Male	130	64.85 ± 11.52	163.64 ± 6.98	24.22 ± 4.01
	Female	173	60.03 ± 11.23	153.64 ± 6.58	25.41 ± 4.39
	Total	303	62.1 ± 11.59	157.93 ± 8.37	24.9 ± 4.27
Total	Male	880	63.66 ± 21.64	161.35 ± 19.55	NA
	Female	1234	56.69 ± 17.23	153.37 ± 14.18	NA
	Total	2114	59.59 ± 19.49	156.69 ± 17.08	NA

Abbreviation: NA, not applicable.

### 3.3 Amount of Food and Beverages Consumed

An FFQ is the most effective tool for assessing habitual dietary intake. In the present study, consumers were requested to estimate the frequency of consumption of specific foods and beverages over an extended period, such as weekly, monthly, and yearly. This type of food consumption survey can acquire more suitable data for assessing long‐term exposure than other survey types, such as dietary records or recalls (Fialkowski et al. 2010). Nowadays, semi‐FFQs are utilized not only to estimate consumption frequency but also to assess the quantity consumed. They are also used to survey the consumption of specific types of food and beverage products (Nirdnoy et al. 2021). The food consumption data presented in this study were obtained using semiquantitative FFQs. The amounts of foods and beverages consumed were calculated as the mean, SD, and PCTL. Table 3 shows the mean, SD, and 97.5th PCTL consumption for each type of food and beverage in each age group, both per capita and for consumers only. These values were used in the calculation of the EDI in the subsequent steps.

**TABLE 3:** Total consumption data for food and beverage products that contain steviol glycosides.

Age group (year)	Category^a^	Per capita (g/kg bw/day)				Consumer only (g/kg bw/day)			
		*N*	Mean	SD	97.5 PTCL	*N*	Mean	SD	97.5 PTCL
3–9	01	220	0.2426	1.1645	4.0876	22	2.4263	2.9309	NA
	04	220	0.0039	0.0378	0	3	0.2865	0.1863	NA
	05	220	0.0009	0.0066	0.0058	10	0.0190	0.0261	NA
	06	220	0	0	0				
	11	220	0.001	0.0133	0.0007	6	0.0372	0.0781	NA
	12	220	0.0240	0.1138	0.3304	31	0.1701	0.2625	NA
	13	220	0.0240	0.2222	0.0745	5	1.0558	1.1578	NA
	14	220	1.7817	5.0884	17.4268	118	3.3218	6.5805	26.2095
	15	220	0.0009	0.0126	0	1	0.1875	—	NA
10–18	01	265	0.1209	0.5352	1.5811	38	0.8431	1.1909	NA
	04	265	0.0018	0.0225	0	2	0.2338	0.1600	NA
	05	265	0.0008	0.0093	0.0005	6	0.0339	0.0571	NA
	06	265	0	0	0				
	11	265	0.0003	0.0024	0.0021	8	0.0103	0.0103	NA
	12	265	0.0132	0.0506	0.1983	45	0.0775	0.1014	0.4283
	13	265	0.0023	0.0299	0	5	0.1243	0.2002	NA
	14	265	1.0866	2.5548	6.8011	144	1.9996	3.1956	11.2821
	15	265	0.0013	0.0108	0.0092	9	0.0373	0.0486	NA
19–39	01	683	0.0987	0.3942	1.0698	112	0.6016	0.8060	3.3247
	04	683	0.0020	0.0376	0	4	0.3415	0.4075	NA
	05	683	0.0015	0.0151	0.0102	25	0.0413	0.0690	NA
	06	683	0	0	0				
	11	683	0.0008	0.0057	0.0093	37	0.0152	0.0200	NA
	12	683	0.0143	0.0653	0.1645	114	0.0858	0.1400	0.4679
	13	683	0.0161	0.1016	0.2040	54	0.2041	0.3063	1.5554
	14	683	0.6225	1.5944	4.9164	326	1.3042	2.1078	6.5300
	15	683	0.0004	0.0053	0	6	0.0510	0.0266	NA
40–59	01	643	0.0891	0.5928	0.7680	71	0.8065	1.6237	7.1047
	04	643	0.0007	0.0146	0	2	0.2329	0.1684	NA
	05	643	0.0009	0.0068	0.0070	31	0.0178	0.0262	NA
	06	643	0	0	0				
	11	643	0.0008	0.0069	0.0038	25	0.0207	0.0292	NA
	12	643	0.0115	0.0454	0.1213	113	0.0655	0.0908	0.3679
	13	643	0.0136	0.0752	0.1791	51	0.1721	0.2116	0.9287
	14	643	0.2833	0.8397	2.7679	234	0.7784	1.2473	4.5578
	15	643	0.0002	0.0022	0	4	0.0270	0.0080	NA
≥ 60	01	303	0.0446	0.2748	0.5750	21	0.6433	0.8580	NA
	04	303	0.0011	0.0104	0	4	0.0802	0.0496	NA
	05	303	0.0005	0.0072	0.0016	8	0.0187	0.0430	NA
	06	303	0	0.0008	0	1	0.0131	NA	NA
	11	303	0.0018	0.0137	0.0124	14	0.0386	0.0531	NA
	12	303	0.0072	0.0553	0.0711	33	0.0660	0.1576	NA
	13	303	0.0013	0.0110	0	6	0.0668	0.0446	NA
	14	303	0.1251	0.4721	1.1372	72	0.5266	0.8566	4.0893
	15	303	0.0004	0.0056	0	2	0.0681	0.0206	NA
Total	01	2114	0.1058	0.5888	1.1374	264	0.8483	1.4681	5.8538
	04	2114	0.0016	0	0	15	0.232	0.236	NA
	05	2114	0.001	0.0105	0.0039	80	0.0266	0.0477	0.1492
	06	2114	0	0.0003	0	1	0.0131	NA	NA
	11	2114	0.0009	0.0084	0.0063	90	0.0214	0.0353	0.1460
	12	2114	0.0133	0.0642	0.1348	336	0.0837	0.1418	0.4457
	13	2114	0.0123	0.1017	0.1200	121	0.2157	0.3714	1.0412
	14	2114	0.6268	2.1916	5.0455	894	1.4823	3.1773	7.2617
	15	2114	0.0005	0.0068	0	22	0.0488	0.0469	NA

Abbreviation: NA, not applicable; there was no value at 97.5th percentile (population was < 40).

^a^ Food and beverages are classified according to the categories specified in Notification of Ministry of Public Health No. 444 B.E. 2566 (2023): 01. Dairy products and analogs. 02. Fats and oils, and fat emulsions. 04. Fruits, vegetables, seaweed, nuts, and seeds. 05. Confectionery. 06. Cereals and cereal products. 11. Sweeteners, including honey (11.6. Tabletop sweeteners sold directly to consumers). 12. Salts, spices, soups, sauces, salads, and protein products. 13. Foodstuffs intended for specific nutritional uses. 14. Beverages. 15. Ready‐to‐eat savories.

### 3.4 Concentrations of Steviol Equivalents

#### 3.4.1 Analysis of Steviol Glycosides and Their Derivatives

The method used to analyze steviol glycosides and their derivatives was previously confirmed and reported by Phungsiangdee et al. (2023). The method displayed significant linearity and sensitivity, as proven by its coefficient of determination (R^2^) values, which ranged from 0.9911 to 0.9990, 0.9939 to 1.0000, and 0.9973 to 0.9999 for typical samples in the beverage, yogurt, and snack categories, respectively. Precision was measured as the % RSD, which ranged from 1.1% to 9.3%. The accuracy of all concentrations was measured as % recovery, which was within the acceptable range of 70%–120%. All the matrix‐matched results of the matrix effect study are presented as percentage recoveries within the accepted range of 80%–120%. Steviol glycosides have a limit of detection of 0.003–0.078 μg/g and a limit of quantitation of 0.011–0.261 μg/g. To ensure the accuracy of the test results, quality controls for the analysis of steviol glycoside levels included % RSD and % recovery. The results indicated that the % RSD and % recovery fell within 0.00%–12.39% and 70.50%–120.93%, respectively (Table 4).

**TABLE 4:** Concentrations of steviol glycoside equivalents in food and beverages in different categories.

Food and beverage categories (number of items in each category)^a^	Steviol equivalent, mg/kg Mean ± SD (min–max)	Precision, %RSD	Accuracy, %recovery
01.0 Dairy products (*n* = 6) ML ranges from 70 to 330 mg/kg	14.03 ± 8.06 (6.18–29.29)	1.33–11.71	70.50–113.45
04.0 Fruits and vegetables (including mushrooms and fungi, roots and tubers, pulses and legumes, and *aloe vera* ), seaweeds, and nuts and seeds (*n* = 4) ML ranges from 40 to 350 mg/kg	37.88 ± 7.55 (33.54–49.19)	0.58–4.03	76.76–91.00
05.0 Confectionery (*n* = 4) ML ranges from 700 to 1100 mg/kg	402.77 ± 337.36 (42.26–735.32)	1.50–4.53	95.05–120.23
06.0 Cereals and cereal products derived from cereal grains, roots and tubers, pulses, legumes, and the pith or soft core of the palm tree (*n* = 1) ML ranges from 100 to 350 mg/kg	134.23 ± 8.36 (125.87–142.59)	0.00–10.50	72.83–120.93
11.0 Sweeteners, including honey (*n* = 10) ML is not specified, GMP is allowed	9964.74 ± 16,958.85 (216.47–50,090.92)	0.86–7.64	81.02–109.03
12.0 Salts, spices, soups, sauces, salads, protein products: (*n* = 9) ML ranges from 30 to 330 mg/kg	38.53 ± 26.78 (4.60–89.05)	2.70–12.39	73.78–109.64
13.0 Foodstuffs intended for specific nutritional uses (*n* = 3) ML ranges from 270 to 2500 mg/kg	16.53 ± 6.43 (10.31–25.39)	0.00–4.82	81.16–106.38
14.0 Beverages, excluding dairy products (*n* = 39) ML ranges from 115 to 200 mg/kg	64.61 ± 156.84 (0.16–886.23)	0.00–10.24	70.00–120.00
15.0 Ready‐to‐eat savories (*n* = 4) ML is 170 mg/kg	87.13 ± 72.56 (1.26–160.31)	0.00–4.72	75.34–117.74

Abbreviation: ML, maximum use level.

^a^ Each sample was analyzed in triplicate. The %RSD and % recovery ranges were obtained from the analysis of nine steviol glycoside derivatives before calculating the steviol equivalents.

#### 3.4.2 Concentrations of Steviol Glycosides in Foods and Beverages

Table 4 shows the concentrations of steviol glycosides, expressed as mean ± SD and min‐max values, with the latter expressed as steviol equivalents. Eighty food and beverage samples were analyzed, with each sample subjected to triplicate analysis. The average concentrations of steviol equivalents in foods in categories 1, 4, 5, 6, 11, 12, 13, 14, and 15 were 14.03 ± 8.06 mg/kg, 37.88 ± 7.55 mg/kg, 402.77 ± 337.36 mg/kg, 134.23 ± 8.36 mg/kg, 9964.74 ± 16,958.85 mg/kg, 38.53 ± 26.78 mg/kg, 16.53 ± 6.43 mg/kg, 64.61 ± 156.84 mg/kg, and 87.13 ± 72.56 mg/kg, respectively. The measured concentrations in foods in nearly all categories, except for four products in category 14, were compliant with the regulatory limits. The four products with steviol glycosides concentrations that exceeded the regulatory limit were instant mixed coffee, instant mixed tea‐honey, instant ginger original formula, and instant ginger strong taste formula, and their steviol glycosides concentrations were 332.42 mg/kg, 419.93 mg/kg, 886.23 mg/kg, and 464.45 mg/kg, respectively. The highest concentration, which was 50,091 mg/kg, was detected in tabletop sweeteners, specifically those in powder form. The concentration profiles of the nine derivatives of steviol glycoside are presented in the Table S2. Ruiz‐Rodriguez, Reglero, and Ibañez (2010) reported that the amounts of steviol glycosides in non‐dairy products such as tea, soy sauce, and soju (a traditional liquor made from starch) range from 0 to 13.7 mg/kg, 0 to 104.6 mg/kg, and 0 to 270.6 mg/kg, respectively, with average values of 0.9 mg/kg, 12.8 mg/kg, and 130.7 mg/kg, respectively. In another previous study, low concentrations of steviol glycosides (1–15 mg/kg) were detected in 12 food categories, including tea, fruit and vegetable beverages, other beverages, snacks, soy sauce, mixed pastes, dressings, sauces, kimchi and pickled vegetables, seasoned fish, and grain‐processed foods, whereas a high concentration (131 mg/kg) was observed in soju (Ha et al. 2013). The levels of steviol glycosides detected in tea products and soy sauce in the two abovementioned studies are lower than the mean levels observed in products in categories 14 (64.61 mg/kg) and 12 (38.53 mg/kg) in the present study.

In this study, the concentration data used for the calculation of dietary exposure included actual measured steviol glycoside concentrations, which were used to calculate the EDI of items consumed by individual consumers. As mentioned previously, the labels of 192 products indicated steviol glycosides were part of the ingredients. For samples that were not analyzed, the average concentration of the food or beverage group was used to calculate exposure to steviol glycosides through consumption of the food or beverage group. This approach may have led to an overestimation of the EDI because it was assumed that all remaining foods or beverages within each category contained the mean concentration of steviol glycosides for that category. However, it provided more refined values than using the maximum use level (ML). It should be emphasized that there are various sources of uncertainty in the calculation of dietary exposure, which may result in the underestimation or overestimation of the results. To identify and qualify underestimation or overestimation in this study, we conducted an analysis of uncertainties according to the criteria described by the European Food Safety Authority (Martyn et al. 2022).

### 3.5 Estimated Dietary Intake of Steviol Glycosides and Characterization of the Risk of Exposure

Actual food consumption data, actual body weight, actual concentration, and mean concentration were used for the refined assessment of exposure in four scenarios. EDI was calculated based on body weight per kilogram, with each person's specific body weight factored into the calculation. The risk associated with exposure to steviol glycoside as steviol equivalents is expressed as a percentage of the ADI. Table 5 shows the EDI of steviol glycosides per capita and for consumers only. The EDI per capita was calculated using the mean and high consumption (97.5th percentile of food consumption) values multiplied by the actual concentration or mean concentration (scenarios 1 and 2). The results indicated that the EDI per capita, for both mean and high consumption, was below the ADI for steviol glycosides. The EDI for consumers only exhibited a similar pattern across all the population groups assessed (scenarios 3 and 4). The 3–9 years age group had the highest EDI (scenario 4) due to the consumption of products in category 14, which accounted for 34.52% of the ADI. The highest EDIs in the 10–18, 19–39, 40–59, and > 60+ years age groups, which were primarily from consumption of foods in category 14, were 14.86%, 8.6%, 6.00%, and 5.39% of the ADI for the age groups, respectively. The highest EDI in the total population, which was 35% of the ADI, was observed in category 11. This is attributable to the large number of products in this category.

**TABLE 5:** Estimated dietary intake of foods and beverages that contain steviol glycosides and characterization of the risk of exposure to steviol glycosides.

Age group (year)	Category	Per capita (mg/kg bw/day)					Consumer only (mg/kg bw/day)				
		*N*	Mean		97.5th percentile		*N*	Mean		97.5th percentile	
			Exposure	% of ADI	Exposure	% of ADI		Exposure	% of ADI	Exposure	% of ADI
3–9	01	220	0.0029	0.07	0.0483	1.21	22	0.0287	0.72	NA	NA
	04	220	0.0001	0.00	0	0	3	0.0109	0.27	NA	NA
	05	220	0.0002	0.00	0.0011	0.03	10	0.0035	0.09	NA	NA
	06	220	0	0	0	0					
	11	220	0.0099	0.25	0.0070	0.18	6	0.3642	9.10	NA	NA
	12	220	0.0012	0.03	0.0170	0.42	31	0.0087	0.22	NA	NA
	13	220	0.0004	0.01	0.0012	0.03	5	0.0175	0.44	NA	NA
	14	220	0.0939	2.35	0.9181	22.95	118	0.1750	4.38	1.3808	34.52
	15	220	0	0.00	0	0	1	0.0108	0.27		
10–18	01	265	0.0014	0.04	0.0187	0.47	38	0.0100	0.25	NA	NA
	04	265	0.0001	0.00	0	0	2	0.0089	0.22	NA	NA
	05	265	0.0001	0.00	0.0001	0.00	6	0.0062	0.16	NA	NA
	06	265	0	0	0	0					
	11	265	0.0030	0.08	0.0205	0.51	8	0.1008	2.52	NA	NA
	12	265	0.0007	0.02	0.0102	0.26	45	0.0040	0.10	0.0220	0.55
	13	265	0	0.00	0	0	5	0.0021	0.05	NA	NA
	14	265	0.0572	1.43	0.3583	8.96	144	0.1053	2.63	0.5944	14.86
	15	265	0.0001	0.00	0.0005	0.01	9	0.0022	0.05	NA	NA
19–39	01	683	0.0012	0.03	0.0126	0.32	112	0.0071	0.18	0.0393	0.98
	04	683	0.0001	0.00	0	0	4	0.0129	0.32	NA	NA
	05	683	0.0003	0.00	0.0019	0.05	25	0.0075	0.19	NA	NA
	06	683	0	0	0	0					
	11	683	0.0080	0.20	0.0913	2.28	37	0.1481	3.70	NA	NA
	12	683	0.0007	0.02	0.0084	0.21	114	0.0044	0.11	0.0240	0.60
	13	683	0.0003	0.01	0.0034	0.08	54	0.0034	0.08	0.0257	0.64
	14	683	0.0328	0.82	0.2590	6.48	326	0.0686	1.72	0.3440	8.60
	15	683	0	0.0006	0	0	6	0.0029	0.0736	NA	NA
40–59	01	643	0.0011	0.03	0.0091	0.23	71	0.0095	0.24	0.0839	2.10
	04	643	0	0.00	0	0	2	0.0088	0.22	NA	NA
	05	643	0.0002	0.00	0.0013	0.03	31	0.0032	0.08	NA	NA
	06	643	0	0	0	0					
	11	643	0.0079	0.20	0.0369	0.92	25	0.2027	5.07	NA	NA
	12	643	0.0006	0.02	0.0062	0.16	113	0.0034	0.09	0.0194	0.49
	13	643	0.0002	0.01	0.0030	0.07	51	0.0028	0.07	0.0154	0.38
	14	643	0.0149	0.37	0.1458	3.65	234	0.0410	1.03	0.2401	6.00
	15	643	0	0.000	0	0	4	0.0016	0.04	NA	NA
≥ 60	01	303	0.0005	0.01	0.0068	0.17	21	0.0076	0.19	NA	NA
	04	303	0	0.00	0	0	4	0.0030	0.08	NA	NA
	05	303	0.0001	0.00	0.0003	0.01	8	0.0034	0.09	NA	NA
	06	303	0	0	0	0	1	0.0018	0.04	NA	NA
	11	303	0.0174	0.44	0.1211	3.03	14	0.3772	9.43	NA	NA
	12	303	0.0004	0.01	0.0037	0.09	33	0.0034	0.09	NA	NA
	13	303	0	0.00	0	0	6	0.0011	0.03	NA	NA
	14	303	0.0066	0.17	0.0599	1.50	72	0.0277	0.69	0.2154	5.39
	15	303	0	0.00	0	0	2	0.0039	0.10	NA	NA
Total	01	2114	0.0012	0.03	0.0134	0.34	264	0.0109	0.27	0.0649	1.623
	04	2114	0.0001	0.00	0	0	15	0.0088	0.22	NA	NA
	05	2114	0.0002	0.01	0.0007	0.02	80	0.0049	0.12	0.0273	0.682
	06	2114	0	0	0	0	1	0.0018	0.04	NA	NA
	11	2114	0.0089	0.22	0.0619	1.55	90	0.2091	5.23	1.4281	35.70
	12	2114	0.0007	0.02	0.0069	0.17	336	0.0043	0.11	0.0212	0.53
	13	2114	0.0002	0.01	0.0020	0.05	121	0.0036	0.10	0.0172	0.43
	14	2114	0.0330	0.83	0.2658	6.65	894	0.0781	1.95	0.3826	9.57
	15	2114	0	0.00	0	0	22	0.0028	0.07	NA	NA

Abbreviation: NA, not available.

This study is the first comprehensive study conducted in Thailand that provides sufficient data on exposure to steviol glycosides, including data on population numbers, actual consumption, and steviol glycoside concentrations, which were used in the calculations. Notably, previous studies did not include information regarding comparisons of changes in exposure to LNCSs, including steviol glycosides. However, a review and summary of some of these previous studies is presented below to provide a context for the use of and exposure to LNCSs in different countries. It should be noted that the data from other countries presented herein are not intended to be directly comparable because the sources of the data used in the calculations are different.

Martyn et al. (2018) conducted a global review of studies on sweetener intake between 2008 and 2018 and reported that the estimated intake of steviol glycosides in Japan in 2012 (age groups: > 1 year; *n* = 4510) was below the ADI (0.119%–0.259% of the ADI), indicating no health concerns (Martyn et al. 2018). A subsequent study conducted in 2016 in Japan revealed that the estimated intake of steviol glycoside in the study population was below the ADI (0.20%–0.47% of the ADI) (Martyn et al. 2018). In these Japanese studies, EDI was calculated using consumption data obtained using the market basket approach, which relies on food consumption data from national dietary surveys, along with concentration data derived from average measurements collected by various government‐ or nationally owned research institutes. These measurements were obtained from samples selected based on recorded dietary consumption patterns (Martyn et al. 2018). In a Korean study, the EDI of steviol glycosides was calculated for all age groups from 1 to > 65 years (*n* = 8081). The results revealed a mean exposure risk of 6.1%–14.3% of the ADI and a high exposure risk of 19.8%–35.2% of the ADI (Ha et al. 2013). In 2017, Kim et al. (2017) examined the EDI of steviol glycosides in the entire Korean population, and their results indicated a mean exposure risk of 4.3% of the ADI. A study conducted in Norway revealed that the consumption of steviol glycosides in the population was high, with high maximum use levels, resulting in exposure to steviol glycosides equivalent to 80% of the ADI. However, the approach used in the study was conservative; thus, the authors concluded that there are no issues with steviol glycoside intake among consumers in the evaluated age groups (VKM 2014). Tier 3 assessment performed in a study conducted in Belgium revealed that the consumption of steviol glycosides by high‐intake consumers (95th percentile) aged 4–6 years old exceeded the ADI by 118.8% (Dewinter et al. 2016). It has been reported that using the maximum permitted steviol glycoside levels for consumed foods as a conservative approach to estimating dietary exposure to steviol glycosides does not reveal a health concern in any age group. Updated studies on exposure to steviol glycosides have been conducted in Brazil (2022), Belgium, and Australia/New Zealand (2023). The results of the Brazilian study indicated that the mean and high EDI of steviol glycosides in the general population were 1.7 mg/kg bw (42.5% ADI) and 5.2 mg/kg bw (131% ADI), respectively (Takehara et al. 2022). In the study conducted in Belgium, the results showed that the mean EDI calculated using actual concentration data was well below the ADI in all analyzed age groups. However, the data obtained in the study were similar to those reported in previous studies: children (aged 3–9 years) had higher exposure values than participants in other age groups (Loco et al. 2023). The refined assessment (refined scenario) performed in the study conducted in Australia/New Zealand indicated that the mean and 90th PCTL dietary exposures in the population groups assessed were between 10% and 15% and 15% and 35% of the ADI, respectively (FSANZ 2023).

The results of the present study and previous studies suggest that exposure to steviol glycosides is not a major health risk. However, an important point to consider is that although consumers may be exposed to several LNCSs simultaneously, the risk of exposure to LNCSs was computed separately in these studies by analyzing exposure to each type of LNCS in relation to its ADI. Various types of LNCSs are present in a large number of items sold in markets. The exact nature of the interactions between these LNCSs and the human body, as well as their potential effects on health, especially in the pediatric population, are unknown (Ruanpeng et al. 2017).

### 3.6 The Main Sources of Exposure to Steviol Glycosides

The main sources of exposure to steviol glycosides are listed in Table 6. Category 14, which does not include dairy products, and categories 11 and 1 were the top three sources, with percentages of 27%–91%, 5%–69%, and 2.3%–4.3%, respectively. The participants in the 10–18 years age group had the highest risk of exposure to steviol glycosides owing to their elevated consumption of beverage products, particularly those in sub‐category 14.1.4.2 (non‐carbonated water‐based flavored drinks). The highest exposure to steviol glycosides in the 60+ age group was through the consumption of products in sub‐category 11.6, specifically tabletop sweeteners sold directly to consumers. Individuals aged 40–59 years had the highest exposure to steviol glycosides through the consumption of products in sub‐category 1.1.4, flavored fluid milk drinks. In the present study, the risks of exposure to steviol glycosides through the consumption of products in the other food categories were below 3% of the ADI.

**TABLE 6:** Food and beverage categories that contribute to steviol glycoside exposure.

Food category number	Food and beverage categories	3–9 years (*N* = 220) (%)	10–18 years (*N* = 265) (%)	19–39 years (*N* = 683) (%)	40–59 years (*N* = 643) (%)	60+ years (*N* = 303) (%)
01	Dairy products and analogs, excluding products in category 02.0	2.75	2.38	2.86	4.31	2.25
05	Confectionery	0.15	0.22	0.64	0.62	0.36
06	Cereals and cereal products derived from cereal grains, roots and tubers, pulses, legumes, and the pith or soft core of the palm tree, excluding bakery wares in category 07.0	0.00	0.00	0.00	0.00	0.02
11	Sweeteners, including honey	9.16	4.86	18.48	31.50	69.10
12	Salts, spices, soups, sauces, salads and protein products	1.05	1.00	1.56	2.18	1.35
13	Foodstuffs intended for specific nutritional uses	0.37	0.06	0.61	0.90	0.09
14	Beverages, excluding dairy products	86.53	91.36	75.74	60.43	26.66
15	Ready‐to‐eat savories	0.00	0.12	0.10	0.05	0.17

In a study conducted in Belgium between October 2019 and February 2020, the primary contributors to exposure to steviol glycosides were products in category 14.1.4 (flavored drinks [26%–49%]), followed by those in category 1.4 (flavored fermented milk products, including heat‐treated products [12%–28%]). The third highest risk of exposure to steviol glycoside was through consumption of products in category 4.2.5.2, which include jams, jellies, marmalades, and sweetened chestnut purée as defined by Directive 2001/113/EC (5%–13%) (Loco et al. 2023). These findings are not consistent with those of the present study. A refined assessment conducted in the study by Food Standards Australia New Zealand revealed that “water‐based flavored drinks” and “coffee (or substitute), tea, herbal infusion, and similar” were the largest contributors to the total risk of exposure to steviol glycosides (19% for each food category) in the Australian population aged ≥ 2 years. For New Zealand children aged 5–14 years and 15 years, the primary contributors were “water‐based flavored drinks” (24%) and “coffee or substitutes, tea, herbal infusions, and similar” (31%), followed by “gravy, sauces, and condiments” (19%) (FSANZ 2023).

### 3.7 Strengths and Limitations of This Study

This is the first published study to conduct a health risk assessment of exposure to steviol glycosides in the Thai population. The major strengths of the present study include conducting a local label survey to identify commercially available products potentially consumed by subjects, conducting a consumption survey using a developed semiquantitative FFQ, and using a validated method for determining steviol glycosides in food and beverage products by UHPLC–ESI‐MS/MS. These strengths in refined, reliable, and representative methodology result in an assessment that closely reflects the actual exposure to steviol glycosides in the population. However, there were some limitations to the study. The first limitation is that a probabilistic risk assessment was not performed due to the complexity of the raw data and the available software. Another limitation was that only commercially available products were selected for the exposure assessment. Ready‐to‐eat foods and beverages that may contain steviol glycosides, sold in local shops, were excluded from the study. Therefore, the results from the exposure assessment may be underestimated.

## 4 Conclusions

The present study was a comprehensive assessment of dietary exposure to steviol glycosides in Thailand, including the evaluation of major contributors to the exposure and assessment of the potential risks of exposure in the population. The findings revealed that steviol glycosides and their derivatives, which are widely used in various Thai foods and beverages, do not pose health risks to the Thai population, as their estimated intake across all scenarios and age groups remains below the ADI. This study fills the information gap regarding the risk of steviol glycoside exposure in Thailand and provides valuable insights necessary for the implementation of regulatory considerations and industry guidelines, emphasizing consumer safety in the consumption of both domestic and exported products. Moreover, the results of this study can serve as a reference for adjusting the maximum daily intake levels for steviol glycosides and their derivatives in accordance with public health regulations. Furthermore, this research can serve as a foundation for informing decision‐making and facilitating ongoing discussions with representatives of relevant industry sectors through public hearings. Overall, the results of this study will significantly contribute to the understanding and management of the risks associated with steviol glycoside exposure in the Thai population.

## Author Contributions

**Pharrunrat Tanaviyutpakdee:** conceptualization (equal), formal analysis (equal), funding acquisition (equal), methodology (equal), resources (equal), supervision (equal), writing – original draft (equal), writing – review and editing (equal). **Pimpuk Phitwongtaewan:** data curation (equal), project administration (equal), writing – original draft (equal), writing – review and editing (equal). **Yollada Phungsiangdee:** data curation (equal), investigation (equal), validation (equal), writing – original draft (equal), writing – review and editing (equal). **Weeraya Karnpanit:** formal analysis (equal), methodology (equal), writing – original draft (equal), writing – review and editing (equal). **Pranee Phattanakulanun:** investigation (equal), writing – original draft (equal). **Thipaporn Yomvachirasin:** data curation (equal), writing – original draft (equal), writing – review and editing (equal). **Songsak Srianujata:** conceptualization (equal), methodology (equal), writing – review and editing (equal).

## Ethics Statement

The study protocols and procedures were approved by the Committee for Research Ethics (Social Sciences), Faculty of Social Sciences and Humanities, Mahidol University (MUSSIRB No. 2020/093 (B1)) on February 29, 2020.

## Consent

All authors approved to participate in this research work and in the manuscript.

## Conflicts of Interest

The authors declare no conflicts of interest.

## Supporting information


Appendix S1

## Data Availability

All data that support the findings of this study are available upon request.
