# Correction to “Risk of Exposure to Steviol Glycosides Through Consumption of Foods and Beverages: A Survey of the Thai Population”

**DOI:** 10.1002/fsn3.71306

**Published:** 2025-12-11

Tanaviyutpakdee, P., P. Phitwongtaewan, Y. Phungsiangdee, W. Karnpanit, P. Phattanakulanun, T. Yomvachirasin, and S. Srianujata. 2025. “Risk of Exposure to Steviol Glycosides Through Consumption of Foods and Beverages: A Survey of the Thai Population.” Food Sci Nutr 13: e4681. https://doi.org/10.1002/fsn3.4681.

An incorrect ethical approval date was reported in section 2.3.1 and ethical statement.

The originally published sentence in section 2.3,

“Prior to the collection of food consumption data, the study protocols and procedures were approved by the Committee for Research Ethics (Social Sciences), Faculty of Social Sciences and Humanities, Mahidol University (MUSSIRB No. 2020/093 (B1)) on February 29, 2020.”

should be corrected to

“Prior to the collection of food consumption data, the study protocols and procedures were approved by the Committee for Research Ethics (Social Sciences), Faculty of Social Sciences and Humanities, Mahidol University (MUSSIRB No. 2020/093 (B1)) on April 14, 2020.”

The originally published sentence in the section ethical statement,

“The study protocols and procedures were approved by the Committee for Research Ethics (Social Sciences), Faculty of Social Sciences and Humanities, Mahidol University (MUSSIRB No. 2020/093 (B1)) on February 29, 2020.”

should be corrected to

“The study protocols and procedures were approved by the Committee for Research Ethics (Social Sciences), Faculty of Social Sciences and Humanities, Mahidol University (MUSSIRB No. 2020/093 (B1)) on April 14, 2020.”

We apologize for the errors.

