# Supplementary material for: Risk of Exposure to Steviol Glycosides Through Consumption of Foods and Beverages: A Survey of the Thai Population
Source: Food Sci Nutr. 2025 Jan 9;13(2):e4681. doi: 10.1002/fsn3.4681 (PMC11788491; doi:10.1002/fsn3.4681)
Supplement: Supplementary file 1 — Appendix S1 [file FSN3-13-e4681-s001.docx]

**Supplementary data**

FIGURE S1 Market survey of products with steviol glycoside and their derivatives


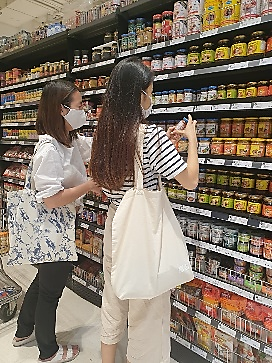

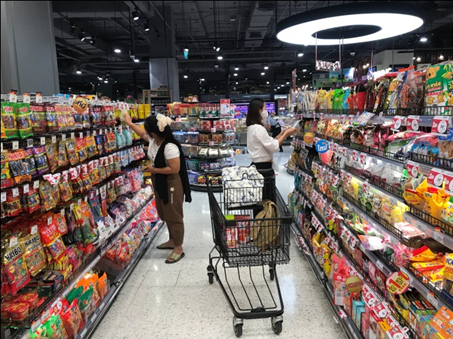


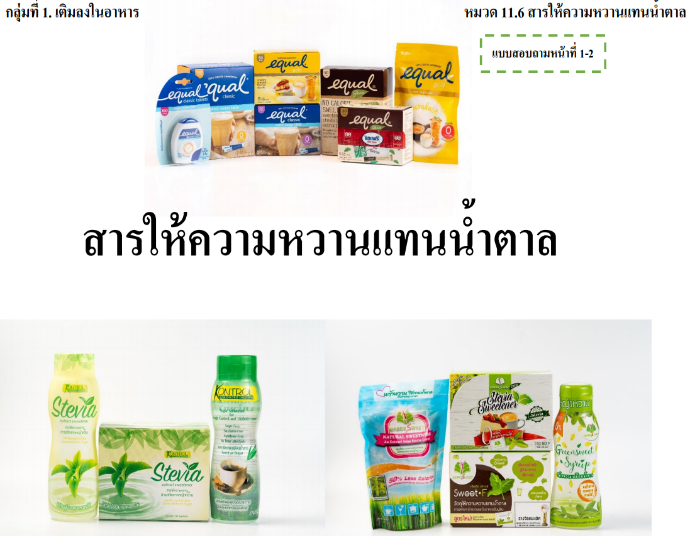

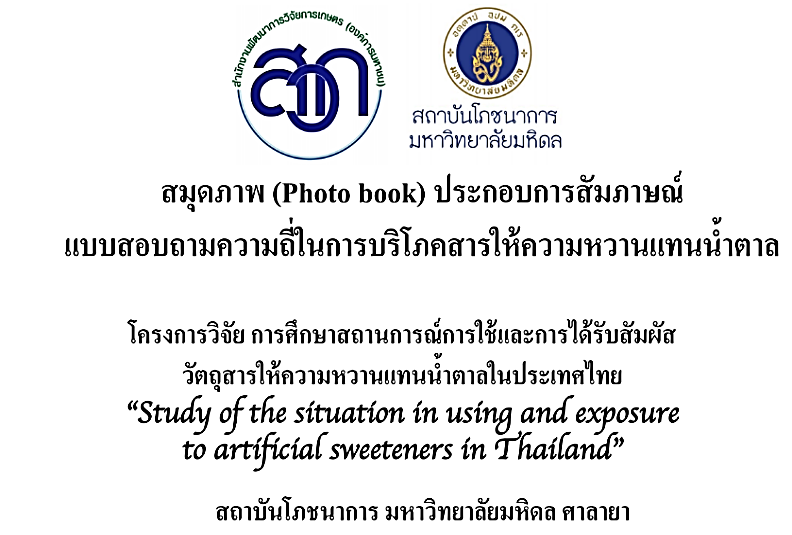
FIGURE S2 Photo book (examples)

FIGURE S3 Questionnaire (personal, KAP questions in Part 1 and semi-quantitative questionnaire in Part 2

Part1


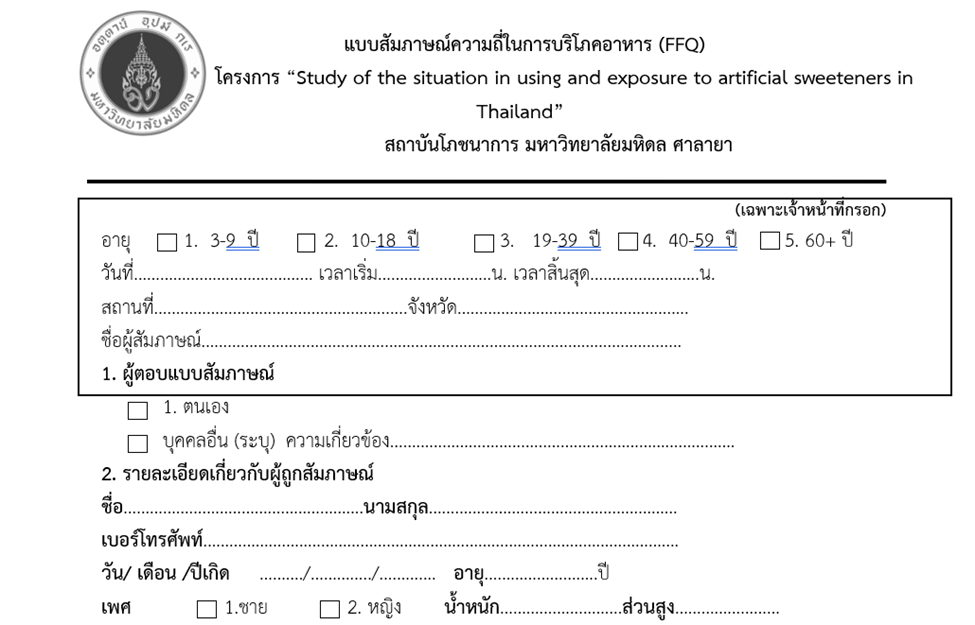


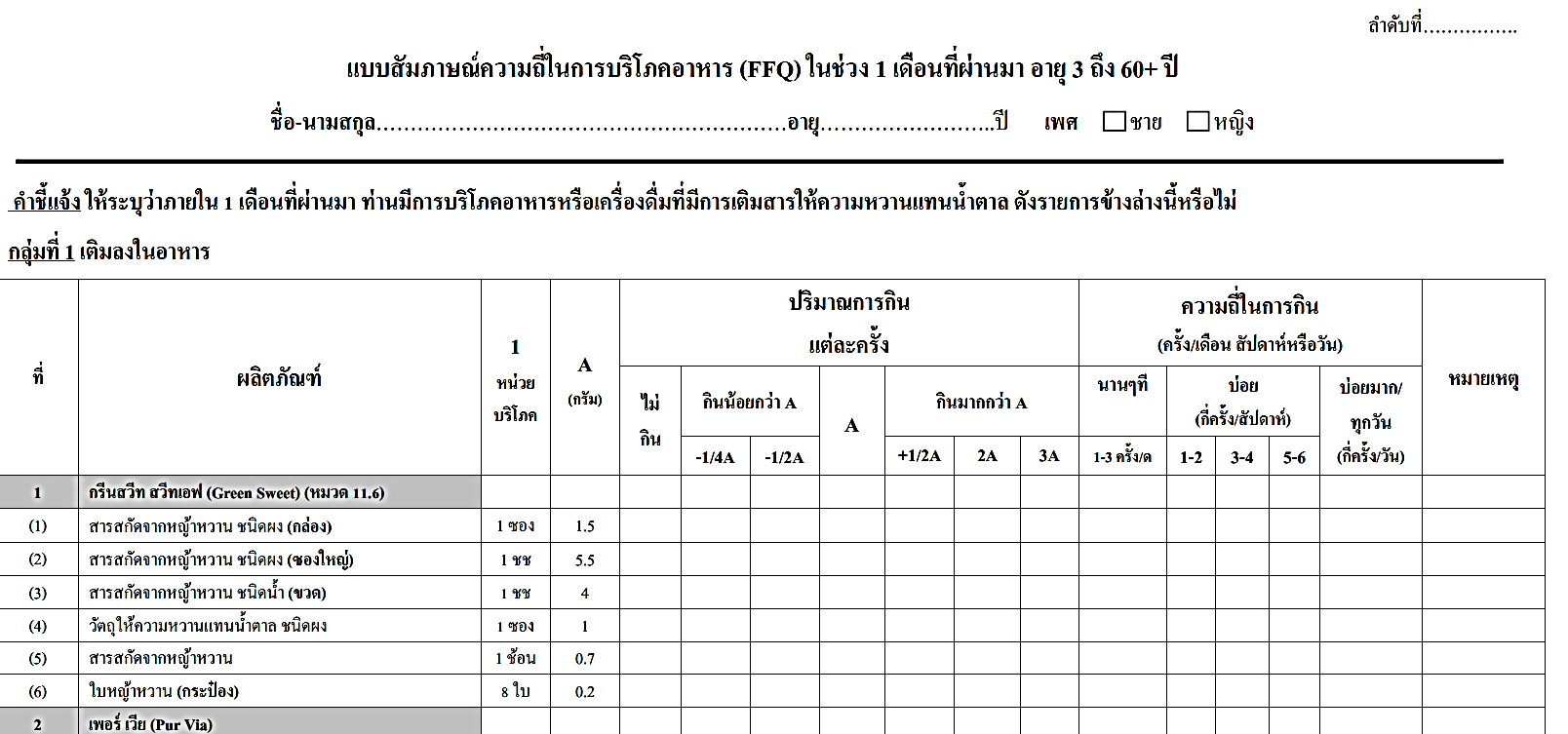


Part 2

TABLE S1 Conversion factor of steviol equivalent (90).

| Steviol glycosides | Conversion factor |
| --- | --- |
| Steviol | 1.00 |
| Stevioside | 0.40 |
| Rebaudioside A | 0.33 |
| Rebaudioside B | 0.40 |
| Rebaudioside C | 0.33 |
| Rebaudioside D | 0.28 |
| Rebaudioside F | 0.34 |
| Dulcoside A | 0.40 |
| Rubusoside | 0.50 |
| Steviolbioside | 0.50 |

| TABLE S2 The concentration of steviol glycoside in food and beverage categories | | | | | | | | | | |
| --- | --- | --- | --- | --- | --- | --- | --- | --- | --- | --- |
| Analysis | RebA | RebB | RebC | RebD | RebF | Rub | Dul | Stevio | Stebio | Steviol equivalent |
| **01.0 Dairy products (n=11)** | | | | | | | | | | |
| Mean ±SD (mg/kg) (Min-Max) | 12.36±5.81 (6.11 - 23.85) | 0.04±0.12 (0.01 - 0.42) | 0.01 | 0.01 | 0.01 | 0.01 | 0.01 | 1.57±3.27 (0.01 – 11.17) | 0.01 | 14.03±8.06 (6.18 – 29.29) |
| Precision (%RSD) | 1.76 – 13.55 | 0.00 - 1.03 | 0.00 | 0.00 | 0.00 | 0.00 | 0.00 | 0.00 – 13.15 | 0.00 | 1.33 – 11.71 |
| Accuracy (%Recovery) | 100.85 - 113.45 | 72.83 – 88.37 | 86.33 – 97.28 | 100.83 – 106.22 | 84.83 – 105.23 | 83.47 – 96.13 | 77.17 – 101.52 | 80.00 – 90.75 | 70.50 – 90.35 |  |
| **04.0 Fruits and vegetables (including mushrooms and fungi, roots and tubers, pulses and legumes, and aloe vera), seaweeds, and nuts and seeds (n=4)** | | | | | | | | | | |
| Mean ±SD (mg/kg) (Min-Max) | 37.65±7.55 (33.31 – 48.96) | 0.03 | 0.03 | 0.03 | 0.03 | 0.03 | 0.03 | 0.03 | 0.04 | 37.88±7.55 (33.54 – 49.19) |
| Precision (%RSD) | 0.58 – 4.03 | 0.00 | 0.00 | 0.00 | 0.00 | 0.00 | 0.00 | 0.00 | 0.00 | 0.58 – 4.01 |
| Accuracy (%Recovery) | 91.01 | 89.79 | 80.15 | 80.63 | 89.75 | 76.76 | 86.74 | 87.71 | 82.82 |  |

|  | | | | | | | | | | |
| --- | --- | --- | --- | --- | --- | --- | --- | --- | --- | --- |
| Analysis | RebA | RebB | RebC | RebD | RebF | Rub | Dul | Stevio | Stebio | Steviol equivalent |
| **05.0 Confectionery (n=4)** | | | | | | | | | | |
| Mean ±SD  (mg/kg) (Min-Max) | 331.30±257.32 (42.03 – 578.01) | 11.11±11.39 (0.03 – 23.32) | 4.43±5.15 (0.03 – 9.80) | 0.91±1.05 (0.03 – 2.11) | 1.91±2.23 (0.03 – 4.37) | 0.03 | 0.03 | 51.30±59.91 (0.03 – 113.82) | 1.76±2.00 (0.04 – 3.84) | 402.77±337.36 (42.26 – 735.32) |
| Precision (%RSD) | 1.55 – 4.55 | 0.00 – 8.28 | 0.00 – 4.35 | 0.00 – 7.98 | 0.00 – 5.06 | 0.00 | 0.00 | 0.00 – 6.49 | 0.00 – 2.21 | 1.50 – 4.53 |
| Accuracy (%Recovery) | 95.05 | 101.43 | 103.67 | 112.77 | 120.23 | 111.49 | 111.30 | 101.35 | 110.29 |  |
| **06.0 Cereals and cereal products derived from cereal grains, roots and tubers, pulses, legumes and pith or soft core of palm tree, excluding bakery wares of food category 07.0 (n=3)** | | | | | | | | | | |
| Mean ±SD  (mg/kg) (Min-Max) | 64.29±2.06 (62.33 – 66.34) | 0.01 | 7.70±0.76 (6.95 – 8.46) | 0.01 | 0.01 | 0.01 | 7.38±0.59 (6.78 – 7.97) | 54.82±4.95 (49.86 – 59.77) | 0.01 | 134.23±8.36 (125.87 – 142.59) |
| Precision (%RSD) | 0 – 10.27 | 0.00 | 0.00 – 13.50 | 0.00 | 0.00 | 0.00 | 0.00 – 15.07 | 0.00 – 12.23 | 0.00 | 0.00 – 10.50 |
| Accuracy (%Recovery) | 100.85 | 72.83 | 120.93 | 106.22 | 92.49 | 96.13 | 101.52 | 90.75 | 90.35 |  |
| Analysis | RebA | RebB | RebC | RebD | RebF | Rub | Dul | Stevio | Stebio | Steviol equivalent |
| **11.0 Sweeteners, including honey (n=12)** | | | | | | | | | | |
| Mean ±SD  (mg/kg) (Min-Max) | 4496.05±6055.39 (112.16 – 21848.89) | 102.84±154.89  (0.03 – 483.97) | 590.11±1282.27  (0.03 – 4000.42) | 34.57±65.21 (0.03 – 232.65) | 119.62±236.38 (0.03 – 743.40) | 43.72±139.66 (0.03 – 486.60) | 134.43±365.65 (0.03 – 1257.45) | 4419.25±9732.13 (0.03 – 28103.47) | 24.16±59.61 (0.04 – 202.85) | 9964.74±16958.85 (216.47 – 50090.92) |
| Precision (%RSD) | 1.10 – 7.84 | 0.00 – 6.50 | 0 – 8.14 | 0.00 – 8.54 | 0.00 – 7.82 | 0.00 – 6.56 | 0.00 – 4.56 | 0.00 – 5.19 | 0.00 - 10.46 | 0.86 – 7.64 |
| Accuracy (%Recovery) | 81.73 – 95.38 | 83.81 – 104.50 | 84.78 – 95.25 | 81.30 – 105.49 | 81.72 – 100.16 | 81.02 – 100.44 | 95.15 – 107.27 | 85.88 – 106.77 | 87.89 – 109.03 |  |
| **12.0 Salts, spices, soups, sauces, salads, protein products: (n=9)** | | | | | | | | | | |
| Mean ±SD  (mg/kg) (Min-Max) | 36.78±25.87 (4.51 – 84.23) | 1.52±1.26 (0.01 – 3.90) | 0.01 | 0.08±0.20 (0.01 – 0.62) | 0.01 | 0.02 | 0.01 | 0.09±0.26 (0.01 – 0.78) | 0.02 | 38.53±26.78 (4.60 – 89.05) |
| Precision (%RSD) | 3.00 – 13.01 | 0.00 – 12.00 | 0.00 | 0.00 – 3.94 | 0.00 | 0.00 | 0.00 | 0.00 - 12.91 | 0.00 | 2.70 – 12.39 |
| Accuracy (%Recovery) | 109.64 | 83.52 | 73.88 | 97.39 | 76.11 | 73.78 | 74.37 | 75.26 | 81.70 |  |

| Analysis | RebA | RebB | RebC | RebD | RebF | Rub | Dul | Stevio | Stebio | Steviol equivalent |
| --- | --- | --- | --- | --- | --- | --- | --- | --- | --- | --- |
| **13.0 Foodstuffs intended for particular nutritional uses (n=4)** | | | | | | | | | | |
| Mean ±SD  (mg/kg) (Min-Max) | 16.30±6.43 (10.08 – 25.16) | 0.03 | 0.03 | 0.03 | 0.03 | 0.03 | 0.03 | 0.03 | 0.04 | 16.53±6.43 (10.31 – 25.39) |
| Precision (%RSD) | 0.00 – 4.90 | 0.00 | 0.00 | 0.00 | 0.00 | 0.00 | 0.00 | 0.00 | 0.00 | 0.00 – 4.82 |
| Accuracy (%Recovery) | 100.71 | 95.33 | 93.63 | 84.98 | 95.27 | 106.38 | 93.20 | 93.33 | 81.16 |  |
| **14.0 Beverages, excluding dairy products: (n=47)** | | | | | | | | | | |
| Mean ±SD (mg/kg) (Min-Max) | 62.85±156.51 (0.003 – 886.16) | 0.79±2.15 (0.01 – 13.48) | 0.14±0.44 (0.01 – 2.32) | 0.02±0.01 (0.01 – 0.03) | 0.02±0.01 (0.01 – 0.03) | 0.03±0.01 (0.01 – 0.03) | 0.02±0.01 (0.01 – 0.03) | 0.69±1.96 (0.01 – 11.52) | 0.03±0.01 (0.01 – 0.04) | 64.61±156.84 (0.16 – 886.23) |
| Precision (%RSD) | 0.00 – 10.41 | 0.00 – 12.66 | 0.00 – 2.72 | 0.03 | 0.00 | 0.00 | 0.00 | 0.00 – 5.23 | 0.00 | 0.00 – 10.24 |
| Accuracy (%Recovery) | 79.39 – 113.45 | 70.00 – 105.21 | 70.88 – 102.00 | 70.36 - 114.52 | 70.88 – 105.23 | 70.00 – 106.38 | 77.34 – 120.00 | 80.00 – 105.32 | 70.00 – 104.95 |  |

| Analysis | RebA | RebB | RebC | RebD | RebF | Rub | Dul | Stevio | Stebio | Steviol equivalent |
| --- | --- | --- | --- | --- | --- | --- | --- | --- | --- | --- |
| **15.0 Ready-to-eat savouries (n=6)** | | | | | | | | | | |
| Mean ±SD (mg/kg) (Min-Max) | 83.68±69.02 (1.18 – 155.36) | 2.97±3.49 (0.01 – 7.99) | 0.01 | 0.16±0.25 (0.01 – 0.62) | 0.01 | 0.02 | 0.01 | 0.26±0.42 (0.01 – 1.02) | 0.03 | 87.13±72.56 (1.26 – 160.31) |
| Precision (%RSD) | 0.00 – 4.82 | 0.00 – 8.15 | 0.00 | 0.00 – 9.39 | 0.00 | 0.00 | 0.00 | 0.00 – 6.48 | 0.00 | 0.00 – 4.72 |
| Accuracy (%Recovery) | 80.85 | 89.02 | 115.04 | 117.74 | 104.90 | 106.81 | 107.57 | 103.23 | 75.34 |  |
